# Comparative Study of SSVEP- and P300-Based Models for the Telepresence Control of Humanoid Robots

**DOI:** 10.1371/journal.pone.0142168

**Published:** 2015-11-12

**Authors:** Jing Zhao, Wei Li, Mengfan Li

**Affiliations:** 1 School of Electrical Engineering and Automation, Tianjin University, Tianjin, China; 2 Department of Computer & Electrical Engineering and Computer Science, California State University, Bakersfield, California, United States of America; 3 Robotics State Key Laborotory, Shenyang Institute of Automation, Chinese Academy of Sciences, Shenyang, China; Duke University, UNITED STATES

## Abstract

In this paper, we evaluate the control performance of SSVEP (steady-state visual evoked potential)- and P300-based models using Cerebot—a mind-controlled humanoid robot platform. Seven subjects with diverse experience participated in experiments concerning the open-loop and closed-loop control of a humanoid robot via brain signals. The visual stimuli of both the SSVEP- and P300- based models were implemented on a LCD computer monitor with a refresh frequency of 60 Hz. Considering the operation safety, we set the classification accuracy of a model over 90.0% as the most important mandatory for the telepresence control of the humanoid robot. The open-loop experiments demonstrated that the SSVEP model with at most four stimulus targets achieved the average accurate rate about 90%, whereas the P300 model with the six or more stimulus targets under five repetitions per trial was able to achieve the accurate rates over 90.0%. Therefore, the four SSVEP stimuli were used to control four types of robot behavior; while the six P300 stimuli were chosen to control six types of robot behavior. Both of the 4-class SSVEP and 6-class P300 models achieved the average success rates of 90.3% and 91.3%, the average response times of 3.65 s and 6.6 s, and the average information transfer rates (ITR) of 24.7 bits/min 18.8 bits/min, respectively. The closed-loop experiments addressed the telepresence control of the robot; the objective was to cause the robot to walk along a white lane marked in an office environment using live video feedback. Comparative studies reveal that the SSVEP model yielded faster response to the subject’s mental activity with less reliance on channel selection, whereas the P300 model was found to be suitable for more classifiable targets and required less training. To conclude, we discuss the existing SSVEP and P300 models for the control of humanoid robots, including the models proposed in this paper.

## Introduction

Brain-Robot Interaction (BRI) refers to the ability to control a robot system via brain signals and is expected to play an important role in the application of robotic devices in many fields [1–3]. Among a variety of robotic devices, humanoid robots are more advanced, as they are created to imitate some of the same physical and mental tasks that humans perform on a daily basis [4]. Achieving control of a humanoid robot is highly challenging, as the typical purpose of a humanoid robot with a full range of body movements is to perform complex tasks such as personal assistance, in which they must be able to assist the sick and elderly, or to perform unsanitary or dangerous jobs. For instance, a subject on a wheelchair can directly control the wheelchair to move [5,6]; while the subject who controls a humanoid robot with full body movements to perform complex tasks needs to activate more behaviors and, especially, has to use live video feedback to telepresence control the humanoid robot in many applications, e.g., the exploration and surveillance in an unknown environment [7].

Methods of acquiring brain signals are classified as either invasive or non-invasive. Non-invasive techniques include magnetoencephalography (MEG), electroencephalograph (EEG), and functional magnetic resonance imaging (fMRI). The most commonly used non-invasive method is the acquisition of EEG signals from electrodes placed on the scalp. This method is inexpensive, easy to use, and provides acceptable temporal resolution. The types of electrical potentials that can be acquired through EEG for the development of control models include motor imagery (MI) potentials, the steady-state visual evoked potentials (SSVEPs), and the P300 potentials. MI potentials, also known as mu/beta rhythms, are induced by the motor cortex through the spontaneous imagining of body movements. Ramos-Murguialday et al. trained a patient to modulate motor imagery potentials to control a neuroprostheses [8]. Typically, an MI-based model delivers limited classifiable states and relatively low classification accuracy; therefore, such a model alone is not commonly used to control a humanoid robot with full body movements; in fact, the sole study in which motor imagery potentials have been used to control the walking gait of a simulated humanoid robot was reported in [9]. To control multiple behaviors of a humanoid robot, Choi et al. combined an MI-based model with SSVEP- and P300-based models [10]. The SSVEP is the potential that naturally responds to visual stimuli at specific frequencies. Tidoni et al. presented an SSVEP-based model for directing a humanoid robot in performing a pick-and-place task [11]. The P300 potential is an event-related potential (ERP) with a positive deflection that is time-locked to auditory or visual stimuli. Bell et al. described a P300-potential-based method for the selection of a target toward which to direct a humanoid robot [12]. However, there is a lack of detailed comparative evaluations of both SSVEP and P300 models.

The objective of this work is to use Cerebot, a mind-controlled humanoid robot platform [13–15], to evaluate and compare both SSVEP and P300 models for the on-line control of the walking behavior of a humanoid robot. To this end, we implemented both SSVEP and P300 models in the OpenVIBE programming environment and conducted experiments involving the control of four robot walking behaviors using the SSVEP model and the control of six robot walking behaviors using the P300 model. The experimental results averaged over seven subjects, including those with no prior experience, indicate the following: 1. The SSVEP model achieved an average success rate of 90.3%, an average response time of 3.65 s, and an average information transfer rate (ITR) of 24.7 bits/min for brain signals acquired from channel Oz. 2. The P300 model, for which 5 repetitions per trial were performed, achieved an average success rate of 91.3%, an average response time of 6.6 s, and an average ITR of 18.8 bits/min for the brain signals acquired from the most responsive channel for each individual. 3. For the P300 model, increasing the number of repetitions per trial improved the success rate but slowed the time response; for example, increasing the repetition number from 5 to 8 caused the average success rate to increase to 98.8% but increased the average response time to 10.56 s and decreased the average ITR to 14.1 bits/min.

This paper is organized as follows: the section Cerebot Platform presents the system architecture of the mind-controlled humanoid robot platform, the section SSVEP and P300 Models discusses the implementation of the SSVEP and P300 models, the section Evaluation Studies describes the evaluation procedures for the SSVEP and P300 models, and the section Experimental Results discusses the evaluation results and compares the performance of both models for the telepresence control of a humanoid robot in a task in which the objective was to cause the robot to follow a white lane marked in an office environment using live video feedback.

## Cerebot Platform

We used Cerebot—a mind-controlled humanoid robot platform that consists of a Cerebus^™^ Data Acquisition System and a NAO humanoid robot—to evaluate the SSVEP and P300 models. The Cerebus^™^ Data Acquisition System is capable of recording, pre-processing and displaying bio-potential signals acquired by various types of electrodes. It provides multiple analog I/O signals and digital I/O signals and is capable of recording up to 128 signal channels simultaneously at a sampling rate of 30 kHz with 16-bit resolution. Its software development kits in C++ and MATLAB provide users with the ability to easily design experimental procedures. In this study, a NAO humanoid robot with 25 degrees of freedom was used to evaluate the SSVEP and P300 models. The NAO robot was equipped with multiple sensors, including 2 cameras, 4 microphones, 2 sonar rangefinders, 2 IR emitters and receivers, 1 inertial board, 9 tactile sensors, and 8 pressure sensors. Both Choregraphe and C++ SDK environments were available for the creation and editing of movements and interactive robot behavior.

**Fig 1:**
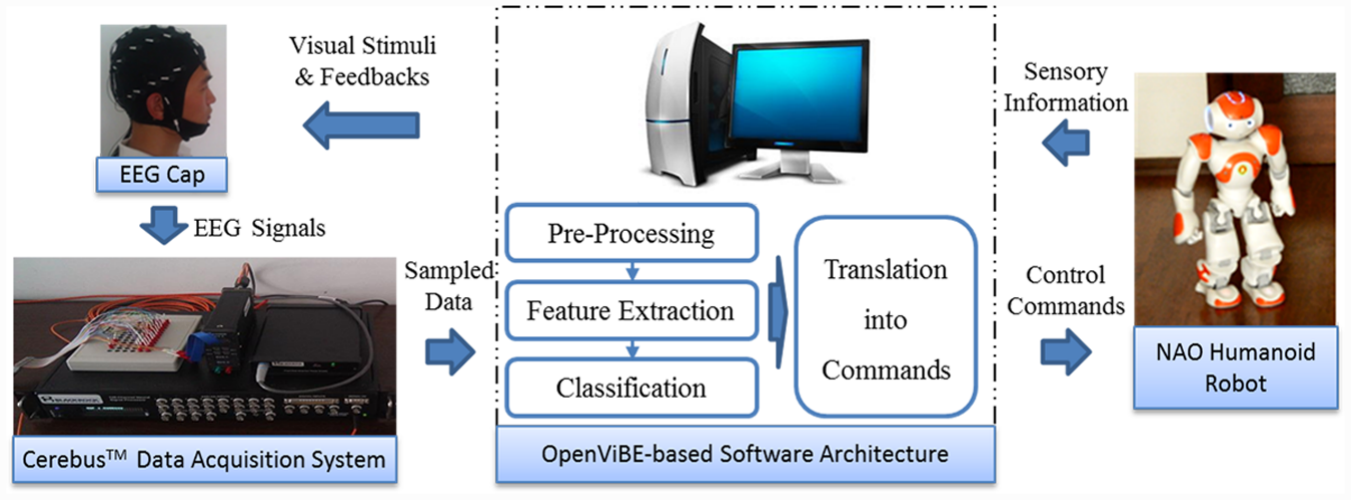
The OpenViBE-based Cerebot structure. Cerebot acquires brain signals via Cerebus^™^, flashes visual stimulus images, and displays live video of the robot’s state and surroundings.

Fig 1 depicts the software architecture of Cerebot for the implementation of control strategies via brainwaves in the OpenViBE environment [13–15]. A number of software programs are integrated into Cerebot, such as OpenGL, OpenCV, MATLAB, Webots, Choregraphe, Central software, and user-developed programs written in C++ and MATLAB [15]. Cerebot acquires brain signals through Cerebus^™^, extracts their features, classifies them based on their patterns, and sends corresponding commands to control the behavior of the humanoid robot via a wireless connection.

## SSVEP and P300 Models

**Fig 2:**
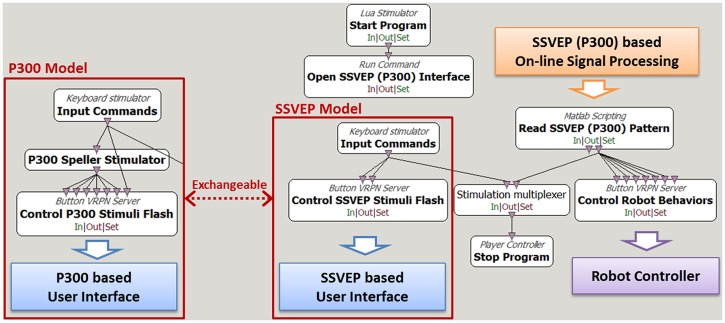
SSVEP and P300 models implemented independently in the OpenViBE programming environment. Execution of the SSVEP or P300 model requires only the switching of the corresponding modules. The modules enclosed in the white boxes are the functions provided by the OpenViBE package, and the arrows indicate data flow paths.

Fig 2 presents the flow diagram for the implementation of the SSVEP and P300 models on Cerebot in the OpenViBE environment. These models consist of modules for the activation of the SSVEP or P300 visual stimulus interface, the on-line processing of the acquired brainwaves, and the control of the behavior of the humanoid robot.

### SSVEP Model

**Fig 3:**
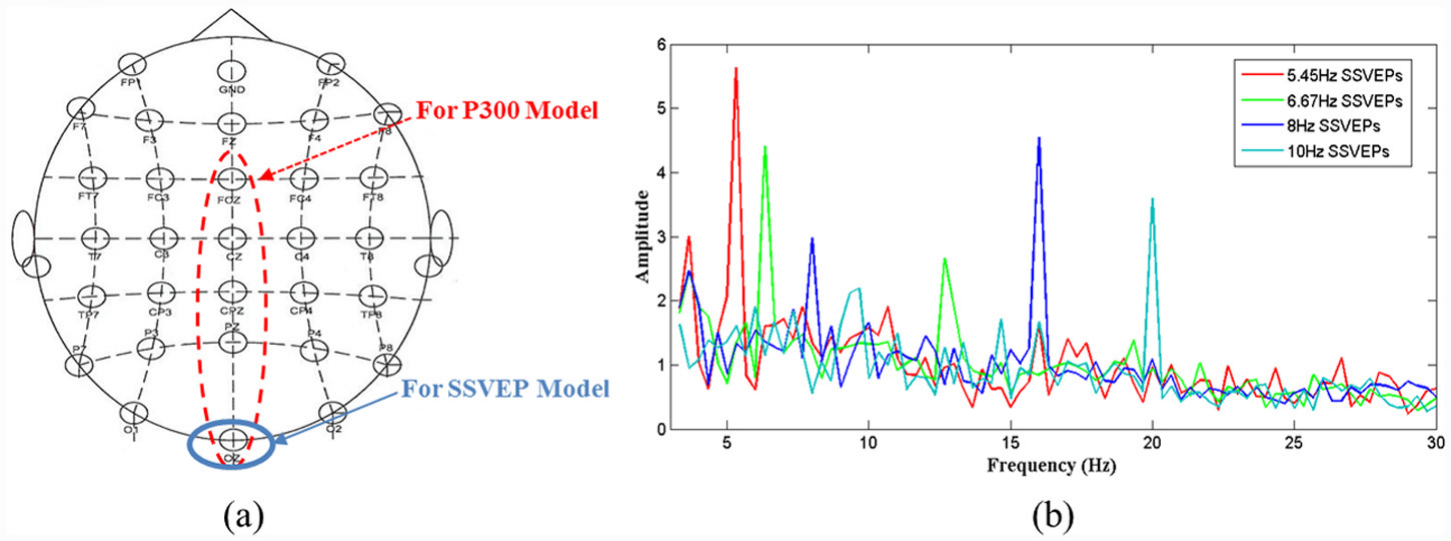
Electrode placement and SSVEP signal spectrum. (a) EEG electrodes placed in accordance with the International 10–20 system. The electrode circled with a blue solid line is the channel in which brainwaves are induced by the SSVEP model, and the electrodes circled with a red dashed line are the prominent channels among which the most responsive channel is selected to acquire the brainwaves induced by the P300 model. (b) SSVEP power spectrum of subject subj1 acquired from channel Oz when the subject was staring at flickering targets modulated at four frequencies.

In 1966, Regan discovered the harmonics of electrical potentials evoked by the flickering of a sinusoidally modulated light using an analog Fourier series analyzer [16]. These types of brainwaves respond to the modulation of visual stimuli at a given frequency and are known as SSVEPs. SSVEPs prominently appear throughout the visual cortex in the occipital region in channels O1, O2 and Oz of the scalp [17], as shown in Fig 3(a). The brain signal *y*_*i*_(*t*) evoked by the *i* ^*th*^ SSVEP stimulus at time *t* is described by [18]

$$y_{i}(t)=\sum_{k=1}^{N_{h}}a_{i,k}\text{sin}\left(2\pi kf_{i}t+\Phi_{i,k}\right)+B_{i,t}\;i=1,2,\ldots ,N$$ (1)

Where *f*_*i*_ is the flickering frequency of the *i* ^*th*^ visual stimulus, *N* is the total number of stimuli, *N*_*h*_ is the number of considered harmonics, *a*_i,k_ and Φ_*i*_,_*k*_ are the amplitude and phase of each sinusoid, and *B*_*i*_,_*t*_ includes noise, artifacts and any components that are irrelevant to the SSVEP response. The SSVEP model implemented on Cerebot consists of two essential modules. The first one is the User Interface for flickering visual stimuli at precise frequencies *f*_*i*_, which elicits brain signals that can be expressed as a number of sinusoids $\sum_{k=1}^{N_{h}}a_{i,k}\text{sin}\left(2\pi kf_{i}t+\Phi_{i,k}\right)$. In this study, the User Interface flickered four images that served as the visual stimuli at 5.45 Hz, 6.67 Hz, 8 Hz, and 10 Hz on a computer monitor [19]. In order to telepresence control the humanoid robot safely, we investigated visual stimuli with the accurate rate over 90%. Considering the available flashing frequencies of the LCD monitor [20] and the influence among harmonic components of SSVEPs [21], we scanned all the possible flashing frequencies from 0 to 60 Hz and tested the classification accuracies of the SSVEP models from 3 to 6 visual stimuli. Table 1 shows that the classification accuracies decreased as the stimuli increased. The 6-class SSVEP model only reached an average accuracy of 83.1%, so the 4-class SSVEP model met the mandatory for control of the four robot walking behaviors: walking forward, walking backward, and turning left and right. The work [22] used the 6-class SSVEP model to control a humanoid robot with a response time of 7.52 s, but it did not explain how to obtain the accuracy and ITR. We could not repeat the tests due to omitting the detailed experimental procedures and the test conditions, but our single channel-based algorithm reached the compatible classification accuracy to the one achieved by the algorithms [23] used for the tests in [22], as listed in Table 1. The second module is the On-line Signal Processing module for the removal of *B*_*i*_,_*t*_ and the extraction of the features of $a_{i,n_{i}}\text{ (}i=1,\;2,\;3,\;4)$, which represent the four stimulus targets, under the constraint $a_{i,n_{i}}>\sigma_{i}$, where *n*_*i*_ represents the most responsive harmonic frequency for the *i* ^*th*^ target and *σ*_*i*_ is the threshold. *n*_*i*_ and *σ*_*i*_ must be calibrated for each subject during an off-line training process because $a_{i,n_{i}}$ strongly depends on the individual and exhibits considerable inherent variability. *n*_*i*_. is determined based on the power spectrum features of the subject.*σ*_*i*_ is calibrated by thoroughly considering the response time and classification accuracy to ensure the following behavior:

$$\sigma_{i}=\left\{ \begin{array}{l} \leq a_{i,n_{i}},\;\text{when}\;\text{subject}\;\text{is}\;\text{staring}\;\text{at}\;\text{the}\;i^{th}\;\text{target}\\ >a_{i,n_{i}},\;\text{when}\;\text{subject}\;\text{is}\;\text{at}\;\text{rest}\;\text{or}\;\text{staring}\;\text{at}\;\text{other}\;\text{targets} \end{array}\right.$$ (2)

Therefore, an experienced researcher will train each subject to shift his/her focal point on an image and to adjust his/her mental state. Fig 3(b) provides an example of the power spectrum features of SSVEP signals.

**Table 1:** Classification accuracy of the SSVEP models from 3 to 6 stimuli.

Model	Classification accuracy (%)			
	3-class	4-class	5-class	6-class
SSVEP	91.9	90.3	88.4	83.1

**Fig 4:**
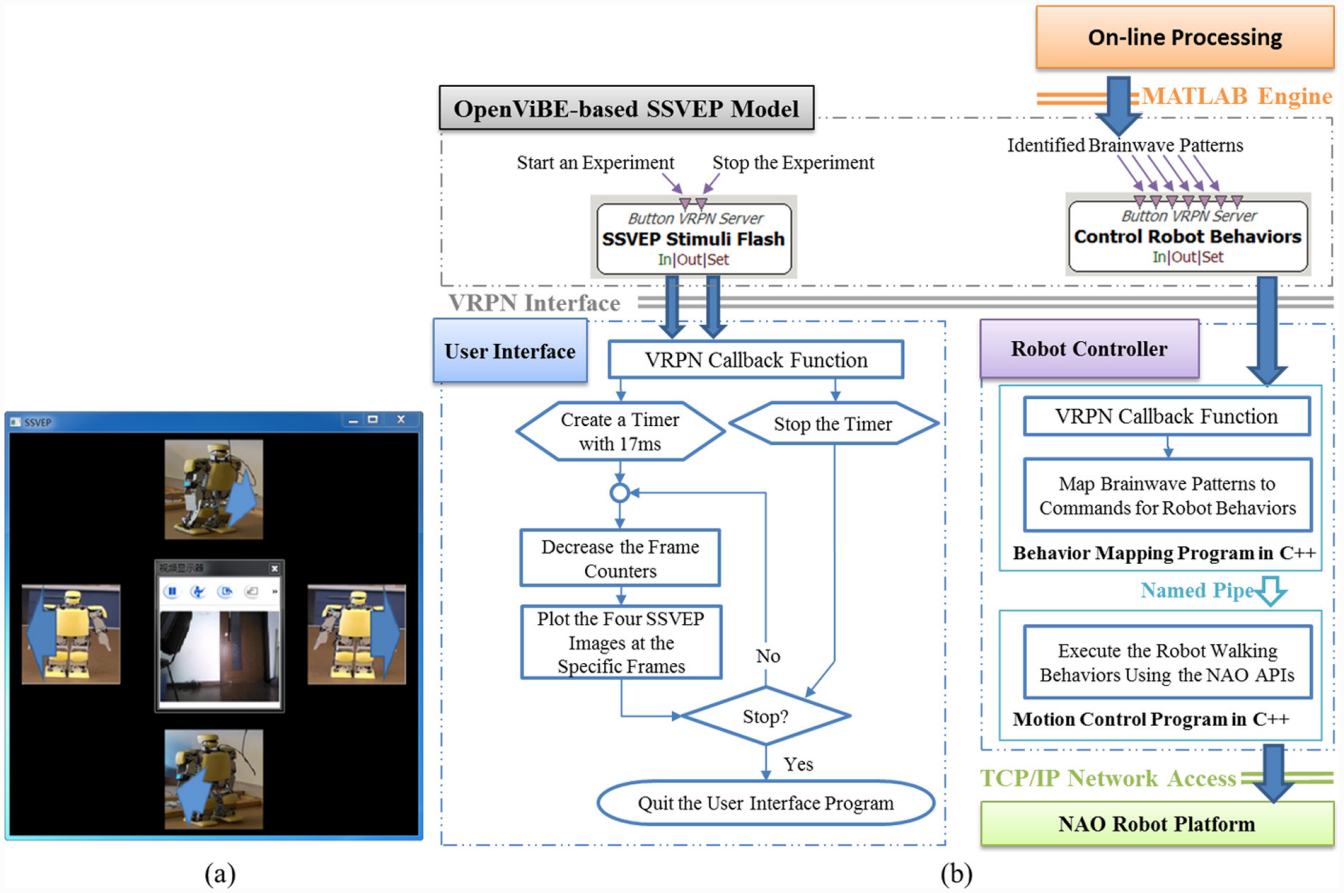
User Interface and its flow diagrams for the SSVEP model. (a) The User Interface for the SSVEP model displays live video in the middle window and flickers four images at four different frequencies on the periphery that represent different humanoid robot behaviors. (b) The flow diagrams describe the User Interface and the Robot Controller for the SSVEP model for an on-line control experiment.

The modules enclosed in the white boxes in Fig 2 are the functions provided by the OpenViBE package, and the arrows indicate the data flow paths. To execute the SSVEP model, the Start Program and Open SSVEP Interface toolboxes initiate the User Interface, which is programmed in C++ and depicted in Fig 4, and the Read SSVEP Pattern toolbox initiates the On-line Signal Processing module via the MATLAB engine. The Input Commands toolbox delivers commands to the Control SSVEP Stimuli Flash toolbox and the Stop Program toolbox to start or end the experimental procedure. The Control SSVEP Stimuli Flash toolbox activates the User Interface, which was displayed in the middle of a 22-inch LCD monitor in this study, as shown in Fig 4(a), to display live video from the camera embedded in the NAO robot via a TCP/IP network. The User Interface simultaneously flashes four robot images on the monitor to serve as visual stimuli; in this study, the monitor had a resolution of 1440×900 pixels and a refresh rate of 60 frames per second. The four images represent four robot behaviors: walking forward, turning right, turning left, and walking backward. Our previous works used several types of humanoid robot images as visual stimuli. In this study, the robot images are used as the visual stimuli to intuitively represent the robot behaviors to be controlled, instead of which type of humanoid robot to be controlled, so the KT-X PC robot images provide very comprehensive information to encode the walking behaviors regardless of robot types, e.g., the KT-X PC robot or the NAO robot. Fig 4(b) shows the flow diagram of the User Interface. The Read SSVEP Pattern toolbox invokes the On-line Signal Processing module, written in MATLAB, which is the key module for the management of an experiment; its functions include reading brain signals from the Cerebus^™^ EEG system and translating them into control commands depending on the received brainwave patterns. The Robot Controller receives the control commands from the Control Robot Behaviors toolbox to activate the corresponding robot behaviors. The Robot Controller incorporates Choregraphe, Webots, and two user-developed programs written in C++, as shown in Fig 4(b). The Robot Controller is able to control either the real NAO robot or a virtual robot via the TCP/IP network. Choregraphe is used to create the NAO robot behaviors, and Webots is used to verify these behaviors through the control of a virtual NAO robot. To end the experiment, the Control SSVEP Stimuli Flash toolbox deactivates the User Interface, and the Read SSVEP Pattern toolbox terminates data collection.

The SSVEP model acquires brain signals at a sampling rate of 1 kHz from channel Oz in the occipital region, filters them using a band-pass filter between 3 and 30 Hz, uses a window of 3 sec in width to segment them, and applies a Fast Fourier Transform (FFT) every 1 sec to calculate their power spectrum A(*t*):

A(t)=|FFT(y((t−3)×S+1:t×S))| (3)

Where *S* is the sampling rate, *y*(*t*×*S*) is the datum sampled at *t* sec, and y((*t*−3) × *S*+ 1: *t* × *S*) is a brainwave segment in the window. The most responsive power spectrum at the $n_{i}{}^{th}$ harmonic frequency for the *i* ^*th*^ SSVEP stimulus target is approximately equal to $a_{i,n_{i}}(t)$ in Eq 1 and is normalized as follows:

$$p_{i,n_{i}}\left(t\right)=\frac{a_{i,n_{i}}\left(t\right)}{\sum_{f=3}^{30}\text{A}\left(t\right)/N_{f}}(i=1,2,3,4)$$ (4)

where $\sum_{f=3}^{30}\text{A}\left(t\right)/N_{f}$ denotes the average amplitude of the spectrum between 3 and 30 Hz. The normalized amplitudes $p_{i,n_{i}}$ of the four frequencies that are used to establish the feature vector are detected when it is above the threshold *σ*_*i*_.

### P300 Model

**Fig 5:**
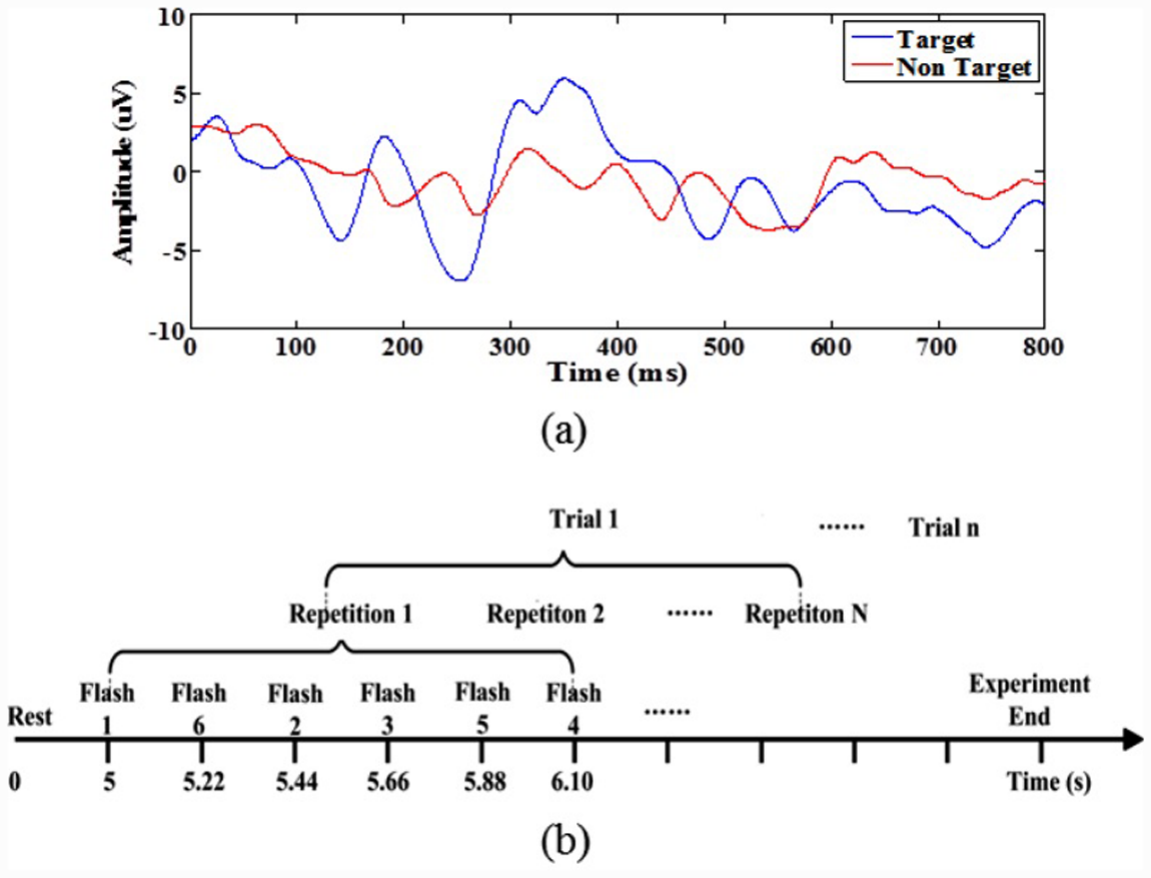
P300 ERPs and our flashing timeline for the P300 model. (a) A P300 potential, which exhibits a large positive deflection at approximately 300 ms, as represented by the blue curve, is recorded in channel Pz when a subject is staring at a flashing target image. (b) Throughout the flashing timeline, the P300 Speller Stimulator toolbox presents the six visual stimuli one by one in a random order.

In 1965, Sutton et al. discovered an electrical potential that exhibited a positive fluctuation within approximately 300 ms after the presentation of an unexpected event (visual, auditory, etc.) [24]. Smith et al. named this potential the ‘P300’ potential based on its polarity and relatively fixed latency [25]. A P300 potential is induced prominently in channels Pz, Fz, and Cz in the midline centroparietal regions, and its latency varies from 300 ms to 800 ms when a set of visual stimuli are presented unexpectedly in a random sequence [26], as shown in Fig 5(a). The feature vector F_*i*_ of this potential for the *i* ^*th*^ target is extracted by capturing the data between 100 and 500 ms and downsampling them.

$${\text{F}}_{i}=downsample\left(\frac{\sum_{j=1}^{N_{r}}y_{i,j}\left(0.1\leq t\leq 0.5\right)}{N_{r}}\right)\;i=1,2,\ldots ,N$$ (5)

Where *y*_i,j_(*t*) is the sampled datum acquired after the presentation of the *i* ^*th*^ P300 target in the *j* ^*th*^ repetition, *N*_*r*_ is the average number of repetitions in a trial, and *N* is the total number of P300 targets. In the P300 model, we set *N*_*r*_ = 5 and *N* = 6. Our study shows that it is easy to implement more stimuli using the P300 model for control of the humanoid robot. However, six walking behaviors, including walking forward, walking backward, shifting left, shifting right, turning left, and turning right, are feasible enough to control a humanoid robot to walk in complex environments, e.g., shifting left or shifting right is able to control the humanoid robot to pass a very narrow path without the need for making a turn, so six P300 stimuli targets are chosen in this study. Then we downsample the brain signals to 20 Hz because representing the feature of P300 responses in a low dimension space allows reducing the computational complexity [27,28]. We were able to down-sample the brain signals from 1000 Hz to 20 Hz [29] according to Shannon’s theorem.

**Fig 6:**
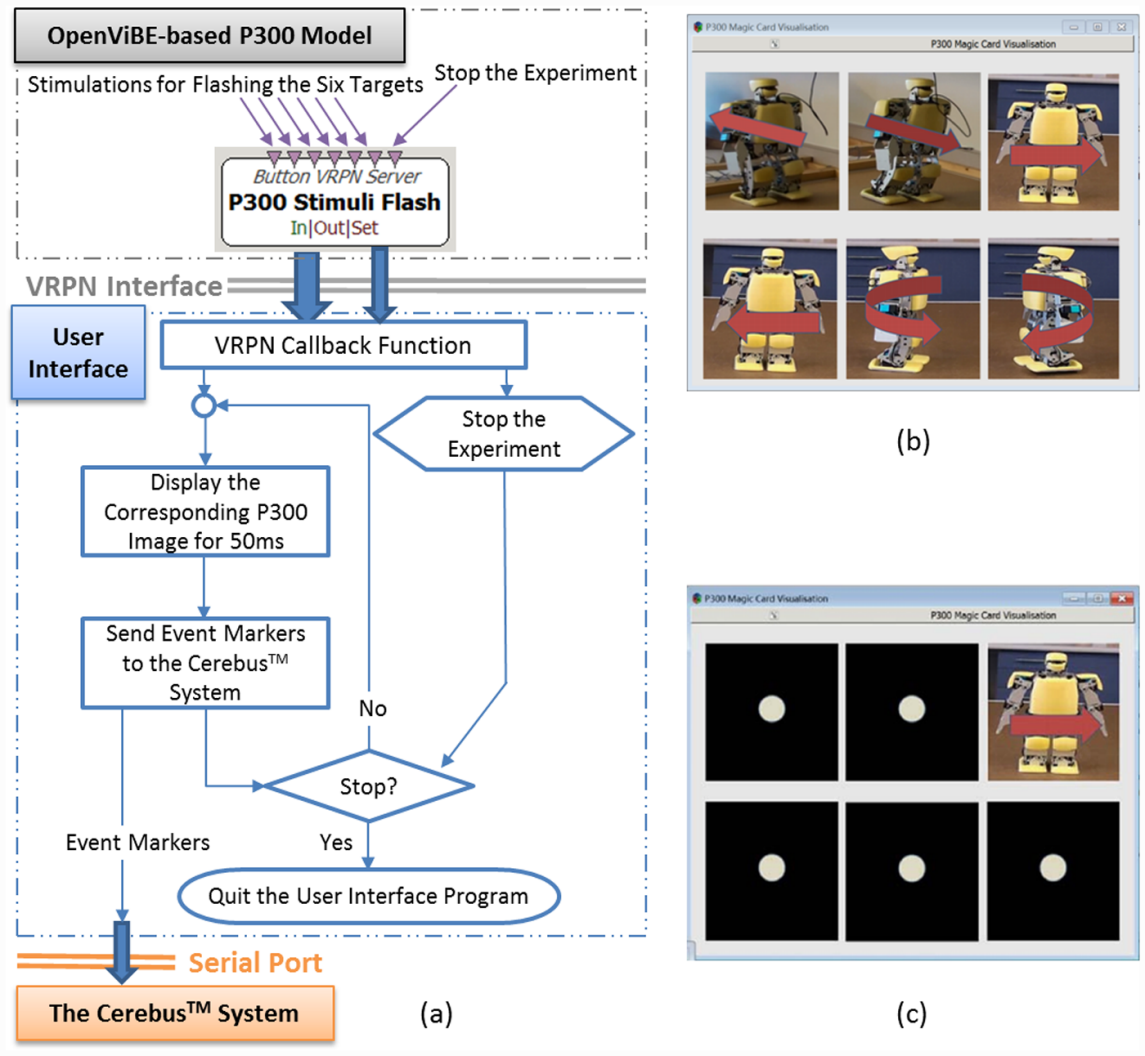
User Interface and its flow diagrams for the P300 model. (a) The flow diagram of the User Interface for the P300 model and its communication with the other modules. (b) The User Interface presents six robot images, each corresponding to different walking behaviors. (c) The User Interface flashes the images one by one in a random order.

Execution of the P300 model in the OpenViBE environment requires only the replacement of the “SSVEP Model” with the “P300 Model” and the switching of the modules Open SSEVP (P300) Interface, Read SSVEP (P300) Pattern, and SSVEP (P300) On-line Signal Processing from the SSVEP model to the P300 model, as shown in Fig 2. The other toolboxes, including Robot Controller, remain unchanged. The P300 model uses the P300 Speller Stimulator provided by the OpenViBE package to load six robot images to serve as visual stimuli and to define their flashing timeline, as shown in Fig 5. The P300 Stimuli Flash toolbox sends the visual stimuli to the P300 User Interface via the VRPN protocol. Fig 6(a) presents the flow diagram for the P300 User Interface, with six robot images representing six robot walking behaviors: walking forward, walking backward, shifting left, shifting right, turning left, and turning right, as shown in Fig 6(b). During a P300 experiment, one repetition consists of flashing each of the six robot images one by one in a random order. Fig 6(c) presents an example in which the shifting-left image is presented while the others are shielded by a black square with a white solid circle. The 1.32 s repetition duration includes all six instances of the presentation of a visual stimulus separated by a 220 ms inter-stimulus interval (ISI), as shown in Fig 5(b).

For the acquisition of P300 potentials with recognizable features, the subject focuses on his/her target stimulus throughout some number of repetitions, constituting a trial. The repetition number of a trial strongly affects the performance of the P300 model. To ensure an objective comparison of the P300 and SSVEP models, we chose to perform the experiments using 5 repetitions per trial. The P300 On-line Signal Processing module processes the acquired brain signals as follows [29,30]. First, the module filters the brain signals using a digital filter with a pass-band of 0.5–26 Hz and divides them into epochs of 500 ms. Second, the module removes the signal drift by subtracting the mean signal value from each epoch and downsamples the signals from 1000 Hz to 20 Hz. Next, the module averages the downsampled signals over all 5 repetitions and uses the FLDA classifier to identify the stimulus target, i.e., the subject’s intention, based on the feature vectors. Finally, the module sends control commands to the Robot Controller to activate the corresponding robot behavior.

## Evaluation Studies

### Subjects

The experiments were performed in an office environment without electromagnetic shielding. The seven subjects (six male and one female, aged 22–29) participated in both the SSVEP and P300 experiments. Among them, subj7, who was the only female subject, was proficient in the P300 experiments but had no prior experience related to the SSVEP experiments; subj1 and subj3 had participated in a number of SSVEP experiments but never in P300 experiments; subj2 had participated in both types of experiments several times; and subj4, subj5, and subj6 had no prior experience related to any of the experiments. All subjects had normal or corrected-to-normal vision and understood the experimental procedures very well. Each subject was seated in a comfortable armchair, 70 cm away from the visual stimuli presented on a 22-inch LCD monitor with a 60 Hz refresh rate. Brain signals were acquired at a sampling rate of 1 kHz using a standard EEG cap with 30 channels, as shown in Fig 3. The ground electrode was placed at FPz on the forehead, and a linked-mastoids reference was used. This project was reviewed and approved by Tianjin medical university general hospital ethics committee, and all subjects gave written consent. Moreover, subj2 also gave a written consent (as outlined in PLOS consent form) to use his facial image in Fig 1 of this article and understood that these case details would be published.

### Evaluation Procedure

The evaluation experiments of the SSVEP and P300 models consisted of off-line calibration process, on-line testing process, and comparative study for closed-loop steering of a real NAO humanoid robot. The off-line calibration process recorded the brain signals of each subject, established his/her feature vectors, and trained the classifier. The coefficients of the SSVEP and P300 models were calibrated during this process, including the configuring of the signal channels, the Cerebus^™^ sampling rate and the classification parameters. Additionally, the subjects with no prior experience used the off-line process to become familiar with the experimental procedure.

**Fig 7:**
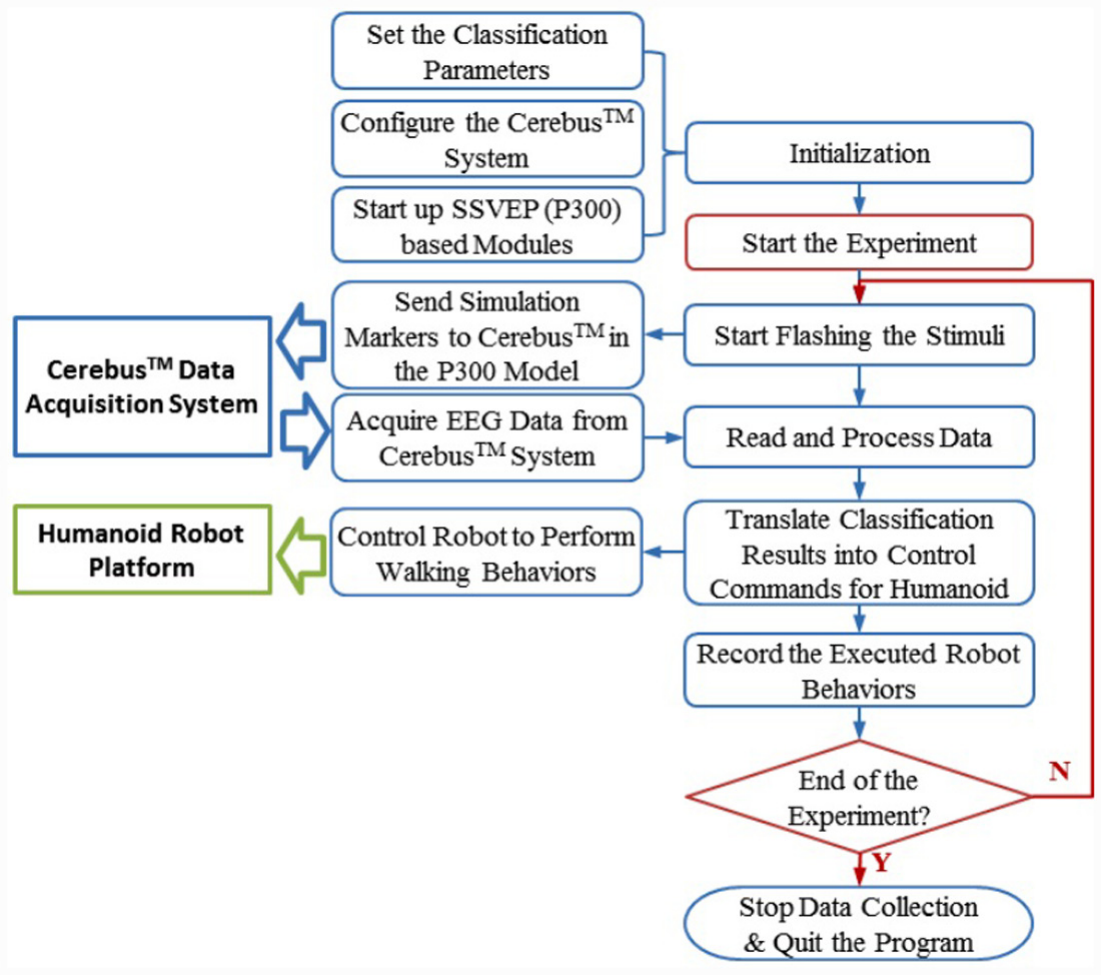
Procedure for the evaluation of the performance of the SSVEP and P300 models in the open-loop experiments for the control of humanoid robot behaviors.

In the on-line testing process, the subjects were requested to control a random sequence of robot walking behaviors through staring at the corresponding visual stimuli. Each subject conducted the experiments using the SSVEP and P300 models respectively to evaluate their performance for the open-loop control of humanoid walking behaviors. Fig 7 presents the flow chart that describes the on-line testing procedure. After the experiment was initiated, the SSVEP or P300 User Interface began flashing the visual stimuli. The P300 User Interface also generated a stimulation marker to indicate when the corresponding visual stimulus was triggered. The On-line Signal Processing module read the brain signals received from Cerebus^™^, processed them, and sent the identified control command to the Robot Controller. We used success rate, response time, and information transfer rate (ITR) to evaluate the performance of the SSVEP and P300 models. The success rate represents the percentage of robot behaviors that were successfully activated. The response time, *T*, represents the time elapsed after the subject received an instruction that the model required to successfully activate the robot behavior. The ITR in units of bits/min is defined in [31].

$$ITR=\frac{p{\text{log}}_{2}\left(p\right)+\left(1-p\right){\text{log}}_{2}\left(\frac{1-p}{N-1}\right)+{\text{log}}_{2}\left(N\right)}{T}$$ (6)

where *N* is the number of defined robot behaviors, *T* is the response time, and *p* is the success rate.

**Fig 8:**
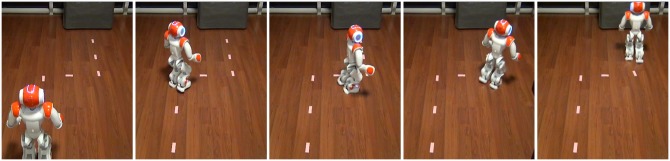
Comparative study of the telepresence control of the humanoid robot with the objective of following a white lane mark in an office environment with live video feedback.

In the comparative experiments of the closed-loop control of the NAO humanoid robot, the objective was to direct the robot to follow a white lane mark with live video feedback using the SSVEP and P300 models respectively, as shown in Fig 8. To objectively evaluate the closed-loop control performance, the subject who achieved the best performance using both the SSVEP and P300 models in the open-loop control evaluations was selected to perform these experiments for 3 repetitions. The subject tried to utilize four robot behaviors defined for the SSVEP model and six robot behaviors defined for the P300 model to control the robot to walk on the path. We used the total execution time and the number of activated behaviors averaged over 3 repetitions of the experiments to evaluate the performance achieved in the closed-loop control.

## Experimental Results

**Fig 9:**
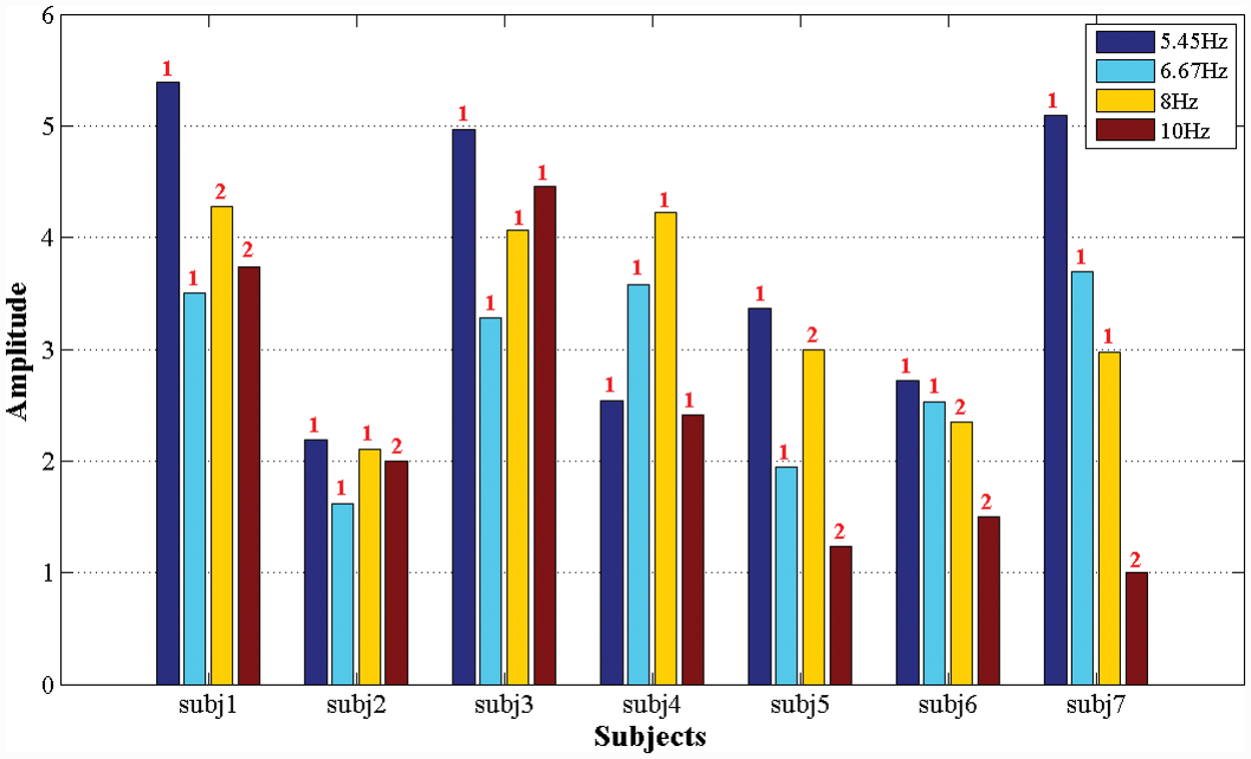
Feature amplitudes at the most responsive harmonic frequency for the seven subjects. These features were induced by the four SSVEP targets flickering at 5.45 Hz, 6.67 Hz, 8 Hz, and 10 Hz. The red numbers above the columns indicate the most responsive harmonic frequencies for each stimulus target and each individual.

Table 2 lists the on-line control results achieved by the seven subjects using the SSVEP model. For all subjects, the brain signals from the single channel Oz were acquired for the evaluations. All subjects presented varying success rates with respect to the four targets flickering at 5.45 Hz, 6.67 Hz, 8 Hz, and 10 Hz, with the exception of subj1. This result can likely be attributed to the inherent differences in their sensitivities to these frequencies. Fig 9 shows the average feature amplitude $p_{i,n_{i}}$ at the $n_{i}{}^{th}$ harmonic frequency that was the most responsive for the *i* ^*th*^ SSVEP stimulus target, as calculated using Eq 4.

**Table 2:** Evaluation results for the control of four robot behaviors using the SSVEP model. The four SSVEP targets flickering at 5.45 Hz, 6.67 Hz, 8 Hz and 10 Hz depicted four robot behaviors: walking forward, turning right, turning left, and walking backward. The overall success rate represents the percentage of successfully activated behaviors among all trials.

Subject	Total trials	Success rates (%) for each stimulus target					Response time (sec)	ITR (bits/min)
		5.45 Hz	6.67 Hz	8 Hz	10 Hz	Overall		
subj1	129	100	100	100	100	100	2.69	44.6
subj2	69	64.3	80.9	90.5	92.3	82.6	3.25	19.5
subj3	33	86.7	100	100	83.3	93.9	3.34	28.2
subj4 ^N^	68	94.4	94.7	100	90.9	95.6	4.46	22.5
subj5 ^N^	72	75	65	90	81.3	77.8	4.24	12.5
subj6 ^N^	31	100	100	62.5	100	90.3	4.26	19.5
subj7 ^N^ ^,^ ^F^	37	100	100	91.7	71.4	91.9	3.33	26.4
Mean±SD		88.6±14.2	91.5±13.6	90.7±13.3	88.5±10.5	90.3±7.7	3.65±0.67	24.7±10.2

^N^New participant in SSVEP experiments.

^F^Female subject.

The following remarks can be made regarding the results. 1. subj1, who understood the SSVEP experiments very well, achieved the highest average success rate of 100%, the shortest average response time of 2.69 s, and the best average ITR of 44.6 bits/min. 2. subj2 achieved a success rate of only 82.6%, even after considerable training, whereas subj4, subj6 and subj7, who were the first-time participants in SSVEP experiments, achieved average success rates of over 90%. These results indicate that subj2’s brain activity is insensitive to the presented SSVEP visual stimuli. 3. The three subjects who were experienced with SSVEP and P300 ERP experiments, subj1, subj2, and subj3, responded to the visual stimuli presented in both experiments much more rapidly than did the subjects who had no prior experience. Interestingly, subj7, who was proficient in P300 experiments, achieved response times comparable to those of the experienced subjects in her first experience with an SSVEP experiment. It is possible that a subject who is proficient in the visual stimuli of one experiment may be able to quickly adapt to the visual stimuli of the other experiment. 4. subj5, who was a first-time participant in SSVEP experiments, underwent a total of 72 trials in two days. On the first day, subj5 became fatigued and his concentration diminished rapidly; therefore, he achieved a success rate of only 72.5% in 40 trials. However, on the second day, his success rate increased to 84.4% in 32 trials.

Table 3 lists the evaluation results obtained in the experiments for the control of six robot walking behaviors using the P300 model: walking forward, walking backward, shifting left, shifting right, turning left, and turning right. Unlike in the SSVEP experiments, in the P300 experiments, brain signals were acquired from five channels, Oz, Pz, CPz, Cz, and FCz, which exhibit considerable differences in their P300 responses to visual stimuli from individual to individual. Therefore, we selected the most responsive channel for each subject, as listed in Table 3, for use in controlling the walking behavior of the robot. All seven subjects, including those with no prior experience, achieved success rates of over 95% using the selected channels. The time required for the classification of a P300 potential is calculated as follows:

$$t=t_{ISI}\times N\times N_{r}$$ (7)

**Fig 10:**
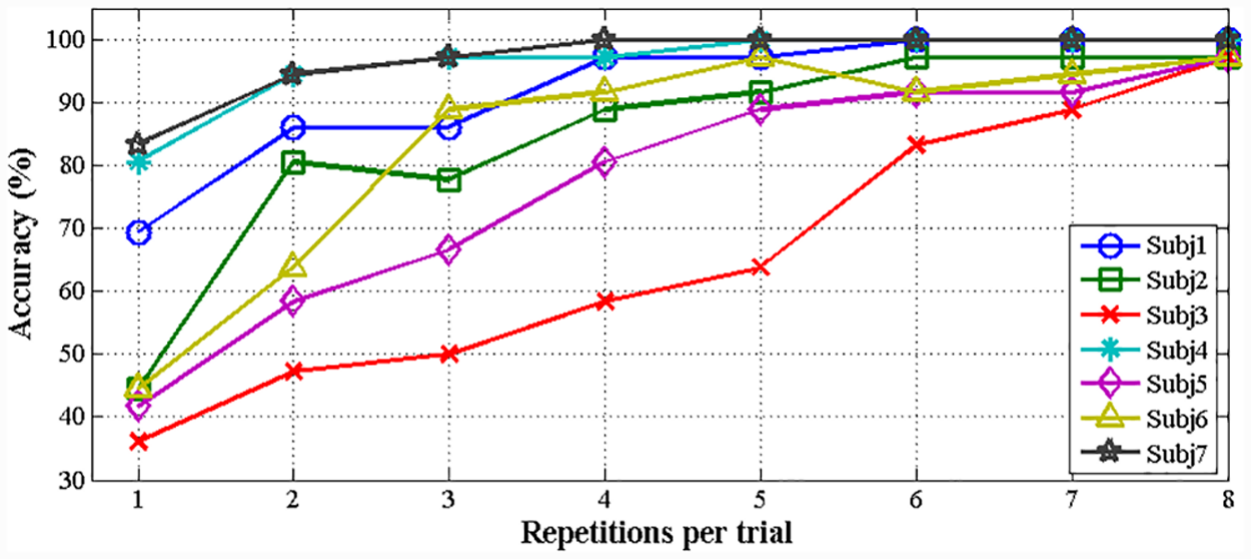
Average accuracy for each subject with different number of repetitions.

Where *t*_*ISI*_ is the inter-stimulus interval of 0.22 s, *N* = 6 is the number of P300 stimulus targets, and *N*_*r*_ is the number of repetitions per trial. Fig 10 presents the average accuracy for each subject vs. the number of repetitions. For *N*_*r*_ = 8, all subjects, including those with no prior experience, achieved success rates of over 95%. Under these conditions, the P300 model requires a response time of 10.56 s to generate a control command. We used a repetition number at which all seven subjects achieved comparable performance using the SSVEP model. We selected 5 repetitions per trial for evaluation because the P300 model with 5 repetitions achieved an average success rate of 91.3%, an average response time of 6.6 s, and an average ITR of 18.8 bits/min for all seven subjects.

**Table 3:** Evaluation results for the control of six robot behaviors using the P300 model with 8 or 5 repetitions per trial.

Subject	Total trials	Most responsive channel	Performance with 8 repetitions		Performance with 5 repetitions	
			Accuracy (%)	ITR (bits/min)	Accuracy (%)	ITR (bits/min)
subj1 ^N^	36	Cz	100	14.7	97.2	21.2
subj2	36	FCz	97.2	13.3	91.7	18.0
subj3 ^N^	36	FCz	97.2	13.3	63.8	7.3
subj4 ^N^	36	Oz	100	14.7	100	23.5
subj5 ^N^	36	Oz	97.2	13.3	88.9	16.6
subj6 ^N^	36	Oz	97.2	14.7	97.2	21.2
subj7 ^F^	36	FCz	100	14.7	100	23.5
Average (Mean±SD)			98.4±1.5	14.1±0.7	91.3±12.8	18.8±5.7

^N^New participant in P300 experiments.

^F^Female subject.

Table 4 summarizes the control performance achieved in the completion of the defined comparative task by subj1, including the total execution time and the number of activated behaviors averaged over three repetitions of the experiments. The average execution times were 96 s and 118 s and the average numbers of output commands were 18.7 and 13.7 for the SSVEP and P300 models, respectively. The P300 model outputs fewer control commands than does the SSVEP model; however, the P300 model requires a longer execution time than does the SSVEP model because the average response time of 8.6 s that is required by the P300 model to output a command is longer than the 5.4 s required by the SSVEP model. Note that for both the SSVEP and P300 models, the average response times for closed-loop control were found to be longer than those for open-loop control. This is because for the closed-loop control experiment, the subject required an additional 2 seconds to output the chosen robot behavior by means of his mental activity based on live video feedback. This additional time of 2 s allowed the subject to make a decision regarding the selection of a suitable robot behavior. The experimental results also show that the P300 model requires the activation of fewer robot behaviors to accomplish the line-following task than does the SSVEP model because the shifting-right and shifting-left behaviors provided by the P300 visual stimuli allow the subject greater flexibility in the control of the walking pattern of the humanoid robot. Table 4 shows that subj1 used 3 TL (turning left) and 4 TR (turning right) behaviors on average to adjust the walking direction of the robot at the two 90-degree corners on the white path when using the SSVEP model, whereas subj1 used 0.7 TL, 1.3 TR and 3 SR (shifting right) behaviors on average when using the P300 model.

**Table 4:** Control performance of both the SSVEP and P300 models in the line-following task. The abbreviations for the robot walking behaviors are as follows: “WF” is walking forward, “WB” is walking backward, “TL” is turning left, “TR” is turning right, “SL” is shifting left, and “SR” is shifting right. The results were averaged over 3 repetitions of the experiments for each model.

Model	Total time(s)	Number of activated behaviors						
		WF	WB	TL	TR	SL	SR	Total
SSVEP	96	10.7	0	3	4.7	N/A	N/A	18.7
P300	118	8.3	0	0.7	1	0	3.7	13.7

## Conclusions and Future Work

In this study, we implemented SSVEP- and P300-based models on Cerebot in the OpenViBE environment and evaluated their performance for both the open-loop and closed-loop control of humanoid robot walking behavior. The evaluation results for the seven subjects can be summarized as follows. 1. The SSVEP model achieved an average success rate of 90.3%, an average response time of 3.65 s, and an average ITR of 24.7 bits/min in the open-loop control of four robot behaviors using the single channel Oz. 2. The P300 model with 8 or 5 repetitions per trial respectively achieved an average success rate of 98.4% or 91.3%, an average response time of 10.56 s or 6.6 s, and an average ITR of 14.1 bits/min or 18.8 bits/min in the open-loop control of six robot behaviors when the most responsive channel for each participant was used. 3. The SSVEP model yields more rapid response to visual stimuli and is nearly independent of channel selection, but the number of the classifiable targets that can displayed on a 22-inch LCD monitor with a 60 Hz refresh rate is limited; meanwhile, the P300 model is capable of providing more classifiable targets and demands even less training, but its response time is slower because it requires flashing the visual stimuli one by one. 4. For both the SSVEP and P300 models, the performance achieved in the closed-loop control task in which the objective was to direct the robot to follow a white line is affected by the live video at which the subject is required to stare to activate the proper mental activity.

Reducing the total number of electrodes may benefit to develop practical BRI devices [32]. In view of controlling the humanoid robot via brain signals, it is essential to develop the algorithms that are easily implemented and run in real-time, so our study aims at comparing both the P300 and SSVEP models using the least number of electrodes, i.e., a signal electrode, a reference electrode, and a ground electrode. Our on-line testing results for 7 subjects show that the SSVEP model achieved an average success rate of 90.3%, and the P300 model with 5 repetitions achieved an average success rate of 91.3%. These accuracy rates meet the requirements on the on-line control of the humanoid robot with live video feedback. Currently, there may be no general superiority of any approach over the others in BCI classification as indicated in [33]. Our SSVEP-based model achieving the compatible performance to the one yielded by the P300-based model used a single channel to telepresence control the NAO robot. The single channel may not be a perfect choice for some BCI systems as the channel layout has to be individualized and the classification accuracies are lower than those using multi-channel techniques [34,35]. However, our research activity aims at the comparative study of the SSVEP and P300 models for the telepresence control of the humanoid robot, which requires the ease of implementation and operates in real-time. For example, the single channel is suitable for our on-going project on education-oriented brain-controlled robot system equipped with a very low-cost EEG device developed by our team because multiple electrodes are not available.

In our study, each subject has to conduct three sessions of experiments. In the first session, the subject conducted an off-line calibration process, which recorded the brain signals for training the classifier. In this case, the subject collected the brain signals of staring at visual stimuli of the P300 and SSVEP models without the need for steering a robot. In the second session, the subject on-line controlled the simulated or physical NAO robot in open loop to randomly activate a sequence of robot behaviors for testing the control success rates. Usually, steering the simulated NAO robot is good for game design projects [36] as the physical robot is unavailable or for the initial practice to get familiar with the brain-controlled NAO robot system in avoiding to damage the real robot. In this study, the subject steered the physical humanoid robot to verify the success rates achieved in the off-line training process. In the third session, that the subject telepresence controlled the physical humanoid robot to perform the line-following task based on live videos was the target of this study. Table 5 summarizes the existing work on the closed-loop control of humanoid robots using the human mind to present a performance comparison based on three criteria (although one or two criteria are lacking for some approaches): success rate, response time, and ITR. Works [9,10,13,22,37,38] report the control of a robot using motor imagery models, which deliver low success rates. Works [9,39,40] report experiments involving the control of a virtual robot; by comparison, controlling a real robot would be much more challenging. Overall, both the SSVEP and P300 models proposed in this paper achieved superior performances compared with those previously reported, as shown in Table 5.

**Table 5:** An overview of the major works concerning the mind-based control of humanoid robots. The abbreviations for the robot walking behaviors are defined as follows: “MI” is a motor imagery model, “ITR” is the information transfer rate, and the P300 models indicated with an asterisk (*) use 5 repetitions per trial.

Publication	Brainwave models	Environment	Task	Evaluation criteria		
				Accuracy (%)	Response time (s)	ITR (bits/min)
Bell et al., 2008^[^ ^12^ ^]^	4-class P300*	Real	Object selection	95	5	24
Li et al., 2011^[^ ^13^ ^]^	3-class MI	Real	Humanoid walking	N/A	N/A	N/A
Chung et al., 2011^[^ ^40^ ^]^	3-class SSVEP	Virtual	Navigation	77.5	N/A	N/A
M Bryan et al., 2011[39], 2012[41]	5-class SSVEP	Virtual & Real	Control of a humanoid arm	N/A	N/A	N/A
Thobbi et al., 2010[22]	2-class MI	Real	Navigation	78.35	N/A	N/A
Finke et al., 2013[38]	P300 and2-class MI	Real	Tasks related to assistance and telepresence	P300: 80 MI: 78	N/A	N/A
Gergondet et al., 2011[42], 2012[43]	4-class SSVEP	Real	Navigation and object selection	N/A	N/A	N/A
Chae et al., 2012[37]	3-class MI	Real	Navigation	80.9	1.84	14.02
Choi et al., 2013[10]	2-class SSVEP, 2-class MI and 4-class P300*	Real	Navigation and object selection	SSVEP: 84.4 MI: 84.6 P300: 91	N/A N/A 5.35	11.6 11.8 15.2
Tidoni et al., 2014[11]	6-class SSVEP	Real	Navigation and pick-and-place tasks	N/A	7.52	N/A
Bouyarmane et al., 2014[9]	2-class MI	Virtual	Moving up stairs	N/A	N/A	N/A
Present work	4-class SSVEP and 6-class P300*	Real	Humanoid walking and navigation	SSVEP: 90.3 P300: 91.3	3.65 6.6	24.7 18.8

In our further research, we will improve the SSVEP and P300 models in the following respects: 1. we will collect brain signals from multiple channels for improved identification of mental activities [35], 2. we will develop effective adaptive algorithms for classifying the visual stimulus targets, and 3. we will explore new visual stimuli to induce brain signals with recognizable features. Jin et al. used multi faces as P300 stimuli to evoke distinct ERPs and Combaz et al. proposed a hybrid BCI Interface by combining both the SSVEP and P300 responses [44,45], but they have not demonstrated their feasibility for on-line control of a humanoid robot with live video feedback yet. We will evaluate their performance for on-line control of the humanoid robot with full body movements. In addition, we will report an evaluation study of a motor-imagery-based model implemented on Cerebot.
